# Assessing Preferences in Patients with Head and Neck Squamous Cell Carcinoma: Phase I and II of Questionnaire Development

**DOI:** 10.3390/cancers12123577

**Published:** 2020-11-30

**Authors:** Pierluigi Bonomo, Alice Maruelli, Calogero Saieva, Katherine Taylor, Susanne Singer, Zaira Patelli, Simon Rogers, Davide Mattavelli, Christian Simon, Florian Scotté, Thiago Bueno de Oliveira, Barbara Murphy, Bethany Andrews Rhoten, Umberto Tassini, Marie Fallon, Ourania Nicolatou Galitis, Noam Yarom, Cristiana Bergamini, Paolo Bossi

**Affiliations:** 1 Radiation Oncology, Azienda Ospedaliero-Universitaria Careggi, 50134 Florence, Italy; bonomop@aou-careggi.toscana.it; 2 Psychology Unit, The Italian League Against Tumors- LILT and Center for Oncological Rehabilitation- CERION-ISPRO, 50134 Florence, Italy; a.maruelli@ispro.toscana.it; 3 Cancer Risk Factors and Lifestyle Epidemiology Unit, Institute for cancer research, prevention and clinical network (ISPRO), 50134 Florence, Italy; c.saieva@ispro.toscana.it; 4 Institute of Medical Biostatistics, Epidemiology and Informatics (IMBEI), University Medical Center Mainz, 55131 Mainz, Germany; kataylor@uni-mainz.de (K.T.); singers@uni-mainz.de (S.S.); 5 Epidemiology and Health services Research, University Cancer Centre, 55131 Mainz, Germany; 6 Department of Medical and Surgical Specialties, Radiological Sciences, and Public Health, University of Brescia, 25123 Brescia, Italy; z.patelli@studenti.unibs.it; 7 Evidence-Based Practice Research Centre (EPRC), Faculty of Health and Social Care, Edge Hill University, St Helens Road, Ormskirk, and Consultant, Head and Neck Centre, Liverpool University Hospital, Lower Lane, Liverpool L97AL, UK; simonn.rogers@aintree.nhs.uk; 8 Unit of Otorhinolaryngology-Head and Neck Surgery, Department of Medical and Surgical Specialties, Radiological Sciences, and Public Health, University of Brescia at ASST Spedali Civili, 25123 Brescia, Italy; davide.mattavelli@unibs.it; 9 Otolaryngology–Head and Neck Surgery, Centre hospitalier universitaire vaudois (CHUV), UNIL, 1011 Lausanne, Switzerland; Christian.Simon@chuv.ch; 10 Interdisciplinary Cancer Course Department, Gustave Roussy Cancer Center, 94805 Villejuif, France; flscotte@gmail.com; 11 Medical Oncology Department, AC Camargo Cancer Center, 01509-010 São Paulo, Brazil; thiago.oliveira@accamargo.org.br; 12 Vanderbilt-Ingram Cancer Center, Vanderbilt Medical Center, Nashville, TN 37232, USA; barbara.murphy@vumc.org; 13 School of Nursing, Vanderbilt University, Nashville, TN 37232, USA; bethany.rhoten@vanderbilt.edu; 14 Italian Federation of Associations of Laryngectomized and Head and Neck Oncological Patients (FIALPO), 37138 Verona, Italy; umbetas@alice.it; 15 Palliative and Supportive Care Group, University of Edinburgh, Edinburgh EH89YL, UK; Marie.Fallon@ed.ac.uk; 16 Clinic of Hospital Dentistry, Dental School, National & Kapodistrian University of Athens, 10679 Athens, Greece; onikolat@dent.uoa.gr; 17 Oral Medicine Unit, Sheba Medical Center, Tel-Hashomer 52621, Israel; noamyar@tauex.tau.ac.il; 18 School of Dental Medicine, Tel-Aviv University, Tel-Aviv 69978, Israel; 19 Head and Neck Medical Oncology, Fondazione IRCCS Istituto Nazionale dei Tumori, 20133 Milan, Italy; cristiana.bergamini@istitutotumori.mi.it; 20 Medical Oncology Unit, ASST Spedali Civili di Brescia and Department of Medical and Surgical Specialties, Radiological Sciences, and Public Health, University of Brescia, 25123 Brescia, Italy

**Keywords:** head and neck cancer, patient preferences, patient priorities, quality of life, multimodal therapies

## Abstract

**Simple Summary:**

Decision-making is often complex and challenging for head and neck cancer. In respect to the disease trajectory and proposed treatment, the patient expression of preferences can be extremely subjective, ultimately depending on several factors such as age at cancer diagnosis, burden of symptoms, closeness to family and availability of caregivers, psychological well-being, and individual cultural, socioeconomic and religious factors. Developing a specific questionnaire to assess patient preferences may allow fostering a more aware patient-centered approach in multidisciplinary management. Through a standardized process for developing questionnaire modules, a 24-item list was generated, laying the ground for further testing and validation on a large scale.

**Abstract:**

Shared-decision making for head and neck squamous cell carcinoma (HNSCC) is challenged by the difficulty to integrate the patient perception of value within the framework of a multidisciplinary team approach. The aim of this study was to develop a questionnaire to assess the preferences of HNSCC patients with respect to the disease trajectory, expected treatment, and toxicities. In accordance with the standardized EORTC Quality of Life Group’s methodology for the development of quality of life modules, a phase 1–2 study was envisaged. Following a systematic review of the literature, a consolidated list of 28 issues was administered through a semi-structured interview to 111 patients from 7 institutions in 5 countries. Overall, “cure of disease”, “survival”, and “trusting in health care professionals” were the 3 most common priorities, being chosen by 87.3%, 73.6% and 59.1% of patients, respectively. When assessing the correlation with the treatment subgroup, the issue of “being thoroughly and sincerely informed about treatments’ efficacy and survival expectation” was highly prevalent in an independent manner (71.4%, 75% and 90% of patients in the follow-up, palliative and curative subgroups, respectively). Based on prespecified scoring criteria, a 24-item list was generated. Pending clinical applicability, further testing and validation of the questionnaire are warranted.

## 1. Introduction

The management of head and neck cancer is to a large extent characterized by a high degree of complexity: standard multidisciplinary treatment is challenged by suboptimal long-term disease control in the curative setting and a dismal outcome in the palliative scenario. Notwithstanding the therapeutic refinement which has taken place in the last 20 years, the prognosis of head and neck squamous cell carcinoma (HNSCC) remains poor, with a cumulative 5-year overall survival (OS) rate of 45–55% in locally advanced disease [1] and a median OS of about one year [2,3,4] in the recurrent/metastatic setting. In addition, radical primary surgery, cisplatin-based concurrent chemo-radiotherapy (CRT), and palliative systemic agents are burdened with a high rate of acute complications, long-lasting treatment-related toxicities [5,6], and marked detriment to patient well-being. In recent years, an increasing interest has been focused on quality of life (QoL) in HNSCC [7,8]. Several patient-reported outcome instruments have been developed [9] to explore the subjective aspects correlated with disease- and treatment-induced side effects, nutritional issues, impairment of social life, lack of perceived daily energy, and financial worries. In all phases of disease trajectory, the assessment of QoL is of paramount importance; however, evaluating QoL domains is different from ascertaining patient preferences. Supported by the best level of evidence available, the multidisciplinary team usually proposes the therapeutic option with the highest cure rate and the safety profile considered tolerable by each patient, as per good clinical practice. However, at present, there is unclear evidence that the cure of disease is the main priority [10,11] for all patients diagnosed with HNSCC. Establishing a value-based framework aimed at integrating patient inclinations into the multidisciplinary team decision process is still an unmet need in head and neck oncology. A holistic approachable to take into consideration patient priorities concerning treatment expectations and to conciliate them with the clinical sphere could lead to better decision-making. The aim of our work was to develop a questionnaire to assess the preferences of patients with HNSCC in regards to treatment and its consequences on several life aspects. In accordance with the standardized European Organization for Research and Treatment of Cancer (EORTC) methodology [12] for the development of QoL modules, our prospective study was designed as a 4-phase process. We report herein the results of phases 1 and 2 of the study, which aim at identifying the most relevant issues (phase 1) and translating them into items (phase 2).

## 2. Results

### 2.1. Identification of QoL Issues Pertaining to Patient Preferences (Phase I)

#### 2.1.1. Literature Search

In accordance with the PRISMA guidelines [13], a systematic review of the literature was conducted on two different databases (Medline and Web of Science). Articles focused on the correlation between patient preferences and treatment-decision making in HNSCC could be included in our analysis. The topic of health-related quality of life was also considered, but only as long as it was addressed within the frame of value-based care. The following MesH terminology was used: head and neck AND cancer or oncol* or neoplasm* or tumor* or tumor* AND patient preferences AND treatment decision-making AND health-related quality of life. Pertinent articles published in English between January 1990 and 31/1/18 (day of the literature search) could be considered, provided that adequate information on patient preferences was reported through structured data (questionnaires). Papers focused on diseases other than HNSCC, case series reporting on less than 20 patients, literature reviews, and consensus statements were not eligible. Data were extracted through a collection sheet by two authors (P.B. and P.B.). Out of a total of 128 records, 11 articles were assessed for eligibility, and 4 of them were finally included in our analysis [10,11,14,15] (Figure S1).

#### 2.1.2. Definition of a Consolidated List of Issues

Two main papers [10,11] reported a detailed description of patient priorities. In view of a slight overlap between them, a merged list of 24 issues was created. Fourteen additional topics were identified from the searched literature, yielding, therefore, a cumulative set of 38 issues. In August 2018, this provisional list was discussed via mail within the steering committee of the study, composed of a multidisciplinary group of international experts in head and neck oncology. In a blinded fashion, they were asked to evaluate the relevance of each issue and to suggest amendments based on their own clinical experience. Principles of clarity and redundancy were also taken into account. After the second round of mail, a consensus was reached among the experts and a consolidated list of 28 issues (Table 1 and Table 2) was finally approved in November 2018. In accordance with a standardized forward-backward procedure of the EORTC Quality of Life Department [12], the definitive table was then translated into the language of the collaborating countries. A minimum number of five different centers and three different languages other than English was mandated by protocol.

#### 2.1.3. Patient Characteristics

In a 4-month time span (December 2018–March 2019), a total of 111 patients were enrolled from 7 institutions worldwide. All patients consented to have a semi-structured interview focused on the consolidated list of issues concerning HNSCC care (Table 2). Patient characteristics are shown in Table 3. Due to regulatory reasons, clinical data were not available for one center (Nashville, 20 patients). The median age of the sample was 63 years (mean 61.7, SD 12.2, range 23–89); most had advanced disease (84% with stage III–IV) but were in good general condition (Karnofsky performance status of >70 in 86.8%). The most common primary HNSCC site was the oral cavity, followed by the oropharynx and nasopharynx (33.7%, 23.5% and 17.8%, respectively). No information on human papilloma virus (HPV) status was retrieved. Overall, the majority of patients enrolled in the study had a high education level (37% >10 years), lived with a partner (61.5%), and benefited from the presence of a caregiver (61.2%). In terms of disease trajectory, subjects undergoing active treatment were slightly more represented than those in surveillance (56.8% and 43.2%, respectively).

#### 2.1.4. Analysis of Patient Preferences

Table 4 reports the distribution of the 28 issues as Likert-type data by simple descriptive statistics. Except for “normal amount of energy” (issue #4), the most frequent answer (mode) was 4 (very much) with a wide distribution, ranging from 94.6% for issue #1 to 33.1% for issue #23. Except for “cure of disease” (#1) and “trusting in health care providers” (#17), the range was 1–4. The reported median values were 3 and 4 for 16 and 12 issues, respectively. Overall, “cure of disease” (#1), “survival-live as long as possible” (#2), and “trusting in health care providers” (#17) were the 3 most common priorities, being chosen by 87.3%, 73.6% and 59.1% of patients, respectively, whereas “normal smell” (#11), “being treated closer to home” (#23) and “social role” (#21) were the 3 least indicated (by only 9.2%, 7.5% and 7.4% of patients, respectively). We then assessed the potential correlation between the pattern of patient preferences and individual characteristics. Several significant differences emerged, mostly in regards to treatment subgroups (Table 5) and treating centers (Table S1). Independent of belonging to the curative, surveillance or palliative cohorts, “normal breathing through mouth and nose” (#13), “burning mouth” (#14), “no depression” (#24) and “being thoroughly and sincerely informed about treatments’ efficacy and survival expectation” (#28) were highly ranked (score of 4) by most patients (Table 5). Overall, a score of 4 was observed most often for issue #28 (71.4%, 75% and 90% of patients in the follow-up, palliative and curative subgroups, respectively; *p =* 0.001). Independent of the geographical location of the treating center, the majority of patients (from 65% to 100%) deemed “cure of disease” (#1), “survival—live as long as possible” (#2) and “being thoroughly and sincerely informed about treatments’ efficacy and survival expectation” (#28) as very much relevant (score of 4; Table S1). Further, by adding together distinct issues into a composite score, we sought to analyze whether a specific cluster of priorities was dependent on the disease trajectory phase. Summating “ability to swallow” (#6), “normal breathing through mouth and nose” (#13) and “burning mouth” (#14), a higher mean value was reported for the curative and palliative subgroups over the follow-up group (10.7, 10.3 and 9.0, respectively; *p =* 0.0001, Kruskal–Wallis test). Compared with patients in follow-up, a heavier burden of treatment-related or disease-induced symptoms may well be expected in curative and palliative settings. The presented data on the composite score #6 + #13 + #14 may reflect this clinical notion with fair internal consistency (Cronbach’s alpha of 0.68). When looking at the summation of “no depression” (#16), “social life” (#24), and “intimacy” (#26), a larger difference was observed between the mean value of the curative subgroup (10.5) and the other two (9.5 and 8.7 in the palliative and follow-up patients, respectively; *p =* 0.009, Kruskal–Wallis test). The internal reliability of this composite score (#16 + #24 + #26: Cronbach’s alpha of 0.74) may lend support to our findings (Table S2). Finally, the consolidated list of issues was presented to 37 health care professionals (HCPs) coming from the recruiting centers with experience in the management of HNSCC. Supplementary Table S3 summarizes the distribution of their results. Overall, “cure of disease” (#1), “ability to swallow (#6)” and “no pain” (#3) were the 3 most common priorities, being chosen by 89.2%, 80.6% and 77.8% of HCPs, respectively.

### 2.2. Creation of a List of Items Pertaining to Patient Preferences (Phase II)

In accordance with the EORTC module development guidelines [12], scoring criteria (Table S4) were taken into account to decide whether a specific issue should be kept or not in the provisional module and ultimately converted into a corresponding item. For each issue, the final score was based on the mean Likert score, the mean relevance for each subgroup and the proportion of patients and HCPs who considered it worthy of inclusion. In 16/28 cases, an intermediate score between 1 and 3 was obtained: based on the aforementioned criteria and clinical expertise, a consensus over whether to keep or delete the issues was reached after an email discussion within the steering committee members in October 2019. By consensus, 4 issues (#14: “burning mouth”; #21: “social role”; #23: “being treated closer to home”; #26: “intimacy”) were removed. At the end of phase 1 of the study, the definitive list of 24 issues on patient preferences was converted into corresponding, easily readable and translatable items (Table 6). For this purpose, an item wording was created from each issue: a final reconciled version was determined within the steering committee. A final check of English wording was performed.

## 3. Discussion

There is a lack of evidence on how to implement value-based care in current head and neck cancer practice [16,17]. Proposed value-frameworks [18,19,20] for shared decision making do not integrate patient-reported outcomes (PROs) together with clinical benefit, safety, and cost [21] of a specific treatment. Notwithstanding the continuous refinement of QoL instruments [22] for HNSCC, it should be borne in mind that within a questionnaire, the relative weight of every single domain is not taken into account by itself. Multiple items are presented into subscales, representing functions or emotions in different fields. These instruments are very informative and useful in describing patient outcomes and comparing treatment arms in clinical trials, although these data do not provide any indications about the value given by a patient on each item or investigated domain. In this perspective, the large heterogeneity of disease behavior, which depends on the primary site involved, and the stage and biology of cancer (such as HPV status), is a confounding, superimposed issue to be considered in head and neck oncology. To the best of our knowledge, our study is the first-ever designed to assess HNSCC patient preferences following a rigorous methodology for QoL module development. In comparison with the evidence available from the literature, the prospective application of a standardized phase I–II process in a multi-national setting reinforces the reliability of the presented data. We acknowledge that the terms “preferences” and “priorities” cannot be perfectly interchangeable; however, for the sake of simplicity, in this manuscript, the terms were employed with the same meaning. Expectedly, we showed that being cured of an extremely burdensome disease such as HNSCC and surviving cancer were indicated as top priorities by the vast majority of the interviewed patients. These results are in line with previous findings from three main studies [10,11,23] that investigated how patients prioritize their preferences on long-term treatment effects based on checklists defined on an ad hoc basis. A prospective observational study from Chicago University [10] showed that 109 of 137 patients (83%) affected by HNSCC ranked survival (“being cured of my cancer” or “living as long as possible”) as their top priority when asked before treatment. Of note, the same choice was less common in elderly patients: only 84% and 43% of subjects older than 65 years placed “being cured of my cancer” and “living as long as possible” in their top 3 rankings compared with 98% and 73% of those younger than 55 years, respectively (*p =* 0.05 and 0.01 for 2 comparisons). However, when looking at how the five single most important individual items were ranked by the patients, a large variability was observed across the cohort. In a cross-sectional study on 300 HNSCC patients interviewed at different time points of disease follow-up, Tschiesner and colleagues [11] showed that surviving cancer was the most relevant priority for 58% of cases. Partly explaining this lower than expected proportion of cancer cure being the main preference, 238 patients (79.3%) had been disease-free for at least 3 years after therapy. The different results from List’s publication [10] may reflect the changing attitude of HNSCC patients over time, shifting more towards financial worries [21] (an area not addressed in the Chicago priority scale) and global functioning. More recently, Windon et al. [23] applied the Chicago priority scale in a prospective cohort study on 122 HNSCC patients surveyed at a median of 7 months after diagnosis: again, cure and survival were the two highest priorities (odds for ranking a top 3 priority at logistic regression; 9.17 and 1.26, respectively), with the latter perceived less relevant with increasing age (odds ratio, 0.72; 95% CI, 0.52–1.00). Interestingly, the expression of preferences did not differ based on HPV status [23,24]. From a more general perspective, a systematic, qualitative review of the literature [25] on HNSCC patient preferences and expectations confirmed that being cured and surviving cancer were top-ranked over functional items in all 20 included studies. Even though large heterogeneity and low-quality data limited the generalizability of these findings, a notable exception was observed in the setting of larynx preservation. Using a time-tradeoff method, it was underlined [26,27] that a consistent subgroup of patients would jeopardize survival in favor of a functional endpoint, namely a better voice outcome. Unlike List’s [10], Tschiesner’s [11] and Windon’s [23] papers, our study additionally draws attention to the central role of patient–doctor relationship in HNSCC: right after cure and survival, trust in health care providers was the most frequent preference, whereas an open communication on disease prognosis and treatment expectations was deemed highly relevant independently from the time point in disease trajectory of the interviewed subjects. Since no formal assessment of decisional regret was performed in our study, it cannot be speculated whether this kind of prioritization was due to disappointment in regards to the individual treatment pathway and patient awareness. Notably, the available evidence suggests that posttreatment dissatisfaction may be low in HNSCC patients: by applying the validated, 5-item Ottawa decision regret scale [28], out of a 0–100 range, the same rate of mild regret was predominant in two studies from different countries (12.5 in a 30 patient-cohort from the UK [29] and 12.7 in a large retrospective analysis on 972 oropharyngeal cancer survivors from MD Anderson Cancer Center [30]; by definition, a threshold of 25 is indicative of moderate to strong regret). Clearly, it can also be hypothesized that our results may suggest how pivotal it is to foster a thorough shared decision-making process in head and neck cancer, aligning patient preferences with the multidisciplinary team vision. Even more than in other primary tumor contexts [31,32], filling the gaps of knowledge in this field should be pursued, as recently highlighted for head and neck surgery [33]. Ideally, the use of instruments such as the questionnaire we are developing may help assess the rate of pretreatment concordance between patients and HCPs [34]. Overall, the variability of preferences among patients in different phases of their treatment journey should be underlined. This reflects the dynamics of expectations, willingness, and fears the patient experiences according to treatment, intent of cure, and burden of experienced symptoms. A comprehensive questionnaire like the one developed here may also help in exploring these complex changes. Finally, the statistically higher relevance of psycho-social aspects reported in the curative subgroup compared with the palliative and follow-up ones in our study may underline the shift occurring in these domains at HNSCC diagnosis or during active treatment, with potential ensuing long-lasting effects [35]. Interestingly, the great concern for intimacy was reported in Windon’s work [23], as well.

The following limitations should be acknowledged when interpreting our results. First, taking into account the relatively small sample size of our cohort and the presence of some missing data, caution should be advised when extrapolating the strength of the associations we found, which should be considered as hypothesis-generating only. For instance, we could not formally ascertain the different potential attitude of patients included in the curative subgroup based on the received primary treatment, being it surgery or definitive chemo-radiation. Second, patient characteristics are somewhat imbalanced in terms of predominant Italian origin (57.6%) and larynx underrepresentation (13.5%). Third, a potential selection bias towards the inclusion of highly educated and compliant patients cannot be minimized. Finally, it should be noted that the outlined results are preliminary and were collected with the intention of developing a new questionnaire through a standard 4-phase process as per EORTC Quality of Life Group methodology: phases III and IV will need to be completed before clinical use of the questionnaire. Phase 3 will be focused on pilot-testing, where HNSCC patients not previously involved in the study will be asked to complete the head and neck cancer patient preferences questionnaire module. Phase 4 will constitute large field-testing to finally validate the questionnaire at an international level, additionally investigating the psychometric properties of the module.

## 4. Materials and Methods

According to the EORTC guidelines for developing questionnaire modules [12], two main phases were envisaged in our prospective study: first, the generation of a list of QoL issues related to patient preferences in HNSCC (phase I); second, the construction of an item list compatible with the common core questionnaire QLQ-C30 by converting the identified issues into corresponding items (phase II). For the sake of clarity, in regards to phase I, the methods used for the systematic review of the literature and the definition of a consolidated list of issues are already described in Section 2.1.1 and Section 2.1.2 together with their corresponding results, respectively.

### 4.1. Identification of Issues Pertaining to Patients′ Preferences (Phase I)

#### 4.1.1. Semi-Structured Interview (Phase I)

Following the EORTC methodology [12], the consolidated list of issues was presented to patients in the form of semi-structured interviews, aiming to ensure content validity. Between December 2018 and March 2019, the patients were asked to go through the list and express the subjective importance of each issue in his/her own experience with respect to their HNSCC care. In particular, their relevance was scored based on a 4-grade scale with the same response categories (scores 1 to 4 corresponding to “not at all”, “a little”, “quite a bit”, and “very much”, respectively). In addition, the patients were encouraged to select the 10 aspects considered as top priorities in their perspective and to suggest up to 3 topics not included in the list. A qualitative content analysis was performed on the additional issues by the steering committee. Within the same time frame and methodology, the consolidated list of issues was also presented to HCPs from the recruiting centers with experience in HNSCC.

#### 4.1.2. Patient Selection (Phase I)

Upon inclusion in the study, the candidate patients had to fulfill the following criteria: signed informed consent; full understanding and ability to complete the questionnaire; performance status of 60–100 according to the Karnofsky scale; age >18 years; histologically confirmed squamous cell carcinoma of the oral cavity, oropharynx, nasopharynx, hypopharynx and larynx; disease stage II, III or IV according to AJCC/TNM 7th edition. Patients in all phases of treatment journey or surveillance could be enrolled, spanning from the date of disease diagnosis. Regarding the patients in active treatment, it was mandated by protocol to state that they had received adequate information on its intent from the treating physician, whether curative or palliative. The follow-up period was defined as all time-points after 6 months from the end of therapy. Overall, enrolled patients were classified in one of the 3 following subgroups: “curative” (either surgery, radiation or CRT-based treatment), “palliative” (chemotherapy-based treatment) or “follow-up”. Patient characteristics such as length of education, living situation and presence or absence of caregiver were recorded at the time of study entry.

#### 4.1.3. Data Collection and Statistical Analysis

All patient questionnaires were centrally collected in the institution of the principal investigator (P.B.). As a first step, each specific issue was separately analyzed as Likert-type data. Thus, individual responses were handled as ordinal data, as this scale is not meant to show the relative magnitude and distance between responses from a quantitative point of view, namely, the degree of relevance of each response can be rated or ranked, but the distance between different scores is not measurable [36,37,38]. Simple statistical analyses were used to describe the distribution of issues, including median and mode for measuring central tendency, and frequency, range and inter-quartile range for measuring variability. A simple frequency statistic was also used to define the 10 issues considered as top priorities by each participant. The association between each issue and selected individual characteristics (center, gender, age, patient subgroup, education, caregiver) was evaluated by appropriate tests for ordinal data, including chi-squared and Kendall Tau B. Second, selected issues were grouped together in order to generate a single “composite score” for each participant in order to provide a quantitative measure of a specific character or personality trait. These summative scales (Likert scale data) were analyzed as interval data by using mean for central tendency and standard deviation for variability, and specific tests (*t*-tests, Pearson’s correlation) to evaluate the association with individual characteristics. Finally, the Cronbach’s alpha was calculated for each composite score as a measure of internal consistency. This coefficient normally ranges between 0 and 1, whereby the closer it is to 1, the greater the reliability of the issues incorporated in the scale [39]. Thus, this test was employed to explore whether the individual issues grouped in the “composite score” were correlated with each other in order to measure the underlying variable. All statistical analyses were performed by using the statistical software SPSS (SPSS Inc, Chicago, IL, USA) for Windows (version 22).

### 4.2. Construction of an Item List (phase II)

Representing the main conclusive result of our phase I–II study of questionnaire development, the final list of patient preferences items is shown in Table 6. For the sake of clarity, the description of how it was generated is provided in Section 2.2.

### 4.3. Ethics Statement

The study was approved by the Ethics Committee of the coordinating center (study code: Priority-HN1/2; protocol number: INT 125/18). Informed consent was obtained from all the enrolled patients.

## 5. Conclusions

Through the EORTC Quality of Life Group’s standardized, two-step process for QoL module development, an initial questionnaire was generated to explore the preferences of patients affected by HNSCC in terms of expectations and feelings on their disease and treatment trajectory. Further testing and validation are required to demonstrate the reliability and clinical applicability of the questionnaire.

## Figures and Tables

**Table 1:** Transition from the initial list of issues to its consolidated version.

#	Issues Retrieved From the Systematic Review	Steering Committee Decision	Consolidated List of Issues
1	Cure of disease	Keep	Cure of disease
2	Survival (live as long as possible)	Keep	Survival (live as long as possible)
3	Pain	Change	No pain
4	Normal amount of energy	Keep	Normal amount of energy
5	Recovery to regular activities (ability to perform daily activities)	Change	Ability to perform daily activities
6	Swallowing solid foods	Merge #6 and #7	Ability to swallow
7	Swallowing liquids	Merge #6 and #7	
8	Natural (normal) voice	Change	Normal voice
9	Unchanged appearance (absence of body mutilation) (cancer consequences not evident to others)	Change	Unchanged appearance of face
10	To be understood	Remove (overlap with normal voice)	
11	Normal chewing	Keep	Normal chewing
12	Normal taste	Keep	Normal taste
13	Normal smell	Keep	Normal smell
14	Moist mouth	Keep	Moist mouth
15	Normal breathing through mouth and nose	Keep	Normal breathing through mouth and nose
16	Tracheostomy	Remove (overlap with normal breathing)	
17	Inflammation of the mouth	Change	Burning mouth
18	Dry mouth	Remove (overlap moist mouth)	
19	Dealing well with anxiety	Change	Cope with anxiety
20	Dealing well with sadness	Change	No depression
21	Stable financial conditions	Remove (overlap)	
22	Trusting in health care providers	Keep	Trusting in health care providers
23	Financial worries for cancer care and follow-up treatments	Change	No financial worries for cancer care/follow-up and for cancer consequences
24	Normal well-being of caregivers	Change	Not being a burden to others
25	Decrease of symptoms	Remove (too generic)	
26	Side effects due to chemotherapy	Remove (redundant and too generic)	
27	Affect day-to-day living activities	Remove (overlap)	
28	Spend time in hospital due to side effects	Change	Spend no time in hospital due to side effects
29	Spend money for the treatment	Remove (overlap)	
30	Social function	Change	Social role
31	Dental health/teeth	Change	Normal dental health
32	Salivation	Remove (overlap)	
33	Being treated closer to home	Keep	Being treated closer to home
35	Frequency of follow up visits	Remove	
36	Frequency of radiological examinations	Remove	
37	Social isolation	Change	Social life
38	Respect for desires and dignity	Keep	Respect for desires and dignity
**Added by the Steering Committee:**			
39			Intimacy
40			Being thoroughly and sincerely informed about treatments’ efficacy and survival expectation

#: number.

**Table 2:** List of issues concerning patient preferences.

#	List of Issues Concerning Patient Preferences
1	Cure of disease
2	Survival (live as long as possible)
3	No pain
4	Normal amount of energy
5	Ability to perform daily activities
6	Ability to swallow
7	Normal voice
8	Unchanged appearance of face
9	Normal chewing
10	Normal taste
11	Normal smell
12	Moist mouth
13	Normal breathing through mouth and nose
14	Burning mouth
15	Cope with anxiety
16	No depression
17	Trusting in health care providers
18	No financial worries for cancer care/follow-up and for cancer consequences
19	Not being a burden to others
20	Spend no time in hospital due to side effects
21	Social role
22	Normal dental health
23	Being treated closer to home
24	Social life
25	Respect for desires and dignity
26	Intimacy
27	Sexuality
28	Being thoroughly and sincerely informed about treatments’ efficacy and survival expectation

#: number.

**Table 3:** Patient characteristics (*n* = 111).

Patient Characteristics		
Center	*N*	%
Brescia (ITA)	32	28.8
Florence (ITA)	10	9.0
Milan (ITA)	22	19.8
Mainz (GER)	10	9.0
Sao Paulo (BRA)	15	13.6
Nashville (USA)	20	18.0
Athens (GRE)	2	1.8
Treatment subgroup *		
Curative	30	34.1
Follow-up	38	43.2
Palliative	20	22.7
Gender *		
Female	27	30.3
Male	62	69.7
Age		
(median 63 y) *		
<70	65	74.7
>70	22	25.3
Education level *		
(years)		
<10	30	35.7
=10	23	27.4
>10	31	36.9
Caregiver *		
No	29	38.2
Yes	47	61.2
Living situation *		
Lives alone	13	14.3
Lives with partner	56	61.5
Lives with other people	6	6.6
Unknown	16	17.6

* values do not sum up to the total number of patients (*n* = 111) due to the fact that some individual information was not available.

**Table 4:** Description of issues as Likert-type data (frequencies, median, mode, range, IQR) and expression of preference (number, percentage) indicated by patients.

Issue	1		2		3		4		5		6		7		8		9		10	
Score	*n*	%	*n*	%	*n*	%	*n*	%	*n*	%	*n*	%	*n*	%	*n*	%	*n*	%	*n*	%
1	0	0	1	0.9	5	4.6	2	1.8	1	0.9	6	5.5	10	9.3	9	8.3	6	5.5	5	4.5
2	2	1.8	1	0.9	14	13	15	13.8	6	5.5	10	9.1	19	17.6	19	17.6	11	10.1	19	17.3
3	4	3.6	10	9.1	32	29.6	48	44	40	36.7	30	27.3	32	29.6	29	26.9	41	37.6	31	28.2
4	104	94.6	98	89.1	57	52.8	44	40.4	62	56.9	64	58.1	47	43.5	51	47.2	51	46.8	55	50
Total	110		110		108		109		109		110		108		108		109		110	
Median	4		4		4		3		4		4		3		3		3		3	
Mode	4		4		4		3		4		4		4		4		4		4	
Range	24		1–4		1–4		1–4		1–4		1–4		1–4		1–4		1–4		1–4	
IQR	0		0		1		1		1		1		2		2		1		1	
Preference	96		81		63		38		63		58		24		26		34		24	
(%)	(87.3)		(73.6)		(58.3)		(34.9)		(57.8)		(52.7)		(22.2)		(24.1)		(31.2)		(21.8)	
**Issue**	**11**		**12**		**13**		**14**		**15**		**16**		**17**		**18**		**19**		**20**	
**Score**	***n***	**%**	***n***	**%**	***n***	**%**	***n***	**%**	***n***	**%**	***n***	**%**	***n***	**%**	***n***	**%**	***n***	**%**	***n***	**%**
1	10	9.2	6	5.5	6	5.5	11	10.1	13	12	12	11.1	0	0	10	9.3	9	8.3	9	8.4
2	17	15.6	21	19.1	7	6.4	21	19.3	21	19.4	22	20.4	5	4.5	13	12.1	12	11	20	18.7
3	34	31.2	27	24.5	24	22	30	27.5	29	26.9	22	20.4	11	10	24	22.4	21	19.3	28	26.2
4	48	44	56	50.9	72	66.1	47	43.1	45	41.7	52	48.1	94	85.5	60	56.1	67	61.4	50	46.7
Total	109		100		109		109		108		108		110		107		109		107	
Median	3		4		4		3		3		3		4		4		4		3	
Mode	4		4		4		4		4		4		4		4		4		4	
Range	1–4		1–4		1–4		1–4		1–4		1–4		2–4		1–4		1–4		1–4	
IQR	2		1		1		2		2		2		0		1		1		2	
preference	10		21		56		17		28		33		65		32		45		29	
(%)	(9.2)		(19.1)		(51.4)		(15.6)		(25.9)		(30.6)		(59.1)		(29.9)		(41.3)		(27.1)	
**Issue**	**21**		**22**		**23**		**24**		**25**		**26**		**27**				**28**			
**Score**	***n***	**%**	***n***	**%**	***n***	**%**	***n***	**%**	***n***	**%**	***n***	**%**	***n***		**%**		***n***		**%**	
1	12	11.1	12	11.3	24	22.6	8	7.5	4	3.7	7	6.6	16		15		4		3.8	
2	26	24.1	19	17.9	26	24.5	19	17.8	14	13	14	13.2	25		23.4		7		6.6	
3	34	31.5	31	29.2	21	19.8	32	29.9	31	28.7	35	33	28		26.2		13		12.3	
4	36	33.3	44	41.5	35	33.1	48	44.8	59	54.6	50	47.2	38		35.4		82		77.3	
Total	108		106		106		107		108		106		107				106			
Median	3		3		3		3		4		3		3				4			
Mode	4		4		4		4		4		4		4				4			
Range	1–4		1–4		1–4		1–4		1–4		1–4		1–4				1–4			
IQR	2		2		2		2		1		1		2				0			
preference	8		13		8		25		28		17		14				51			
(%)	(7.4)		(12.3)		(7.5)		(23.4)		(25.9)		(16)		(13.1)				(48.1)			

IQR: interquartile range.

**Table 5:** Relevance of selected issues by treatment group (*n =* 88). *p*-value from chi-squared or Kendall Tau-b test.

Issue Description	Issue		Curative (*n* = 30) *n* (%)	Follow-Up (*n* = 38) *n* (%)	Palliative (*n* = 20) *n* (%)	*p*-Value from Chi-Squared (Kendall Tau-b)
	*n*	Score				
Ability to swallow	#6	1	2 (6.7)	2 (5.4)	0 (0)	0.008
		2	1 (3.3)	6 (16.2)	3 (15)	
		3	3 (10)	16 (43.2)	4 (20)	
		4	24 (80)	13 (35.2)	13 (65)	
Normal breathing through mouth and nose	#13	1	3 (10)	1 (2.7)	0 (0)	0.009
		2	1 (3.3)	4 (10.8)	0 (0)	
		3	0 (0)	9 (24.3)	7 (35)	
		4	26 (86.7)	23 (62.2)	13 (65)	
Burning mouth	#14	1	2 (6.7)	4 (11.1)	2 (10)	0.041
		2	1 (3.3)	11 (30.6)	4 (20)	
		3	8 (26.7)	10 (27.8)	2 (10)	
		4	19 (63.3)	11 (30.5)	12 60)	
No depression	#16	1	3 (10)	3 (8.6)	4 (20)	0.046
		2	3 (10)	12 (34.3)	1 (5)	
		3	5 (16.7)	7 (20)	2 (10)	
		4	19 (63.3)	13 (37.1)	13 (65)	
Spend no time in hospital due to side effects	#20	1	3 (10)	4 (11.4)	0 (0)	0.029
		2	0 (0)	8 (22.9)	3 (15)	
		3	8 (26.7)	12 (34.3)	4 (20)	
		4	19 (63.3)	11 (31.4)	13 (65)	
Being treated closer to home	#23	1	3 (10)	12 (34.3)	5 (25)	0.06 (0.048)
		2	4 (13.3)	10 (28.6)	5 (25)	
		3	8 (26.7)	6 (17.1)	2 (10)	
		4	15 (50)	7 (20)	8 (40)	
Social life	#24	1	1 (3.3)	3 (8.6)	0 (0)	0.15 (0.025)
		2	1 (3.3)	6 (17.1)	5 (25)	
		3	7 (23.3)	11 (31.4)	6 (30)	
		4	21 (70.1)	15 (42.9)	9 (45)	
Intimacy	#26	1	1 (3.3)	1 (2.9)	2 (10)	0.06 (0.01)
		2	1 (3.3)	6 (17.6)	3 (15)	
		3	6 (20)	15 (44.1)	6 (30)	
		4	22 (73.4)	12 (35.4)	9 (45)	
Being thoroughly and sincerely informed about treatment’s efficacy and survival expectations	#28	1	0 (0)	4 (11.4)	0 (0)	0.001
		2	1 (3.3)	0 (0)	5 (25)	
		3	2 (6.7)	6 (17.1)	0 (0)	
		4	27 (90)	25 (71.4)	15 (75)	

#: number.

**Table 6:** Final list of patient preferences for items resulting from phase II.

#	Items Overall Question: to What Extent do You Consider the Following Items Relevant to You?	1 = Not At All 2 = A Little 3 = Quite A Bit 4 = Very Much
1	Cure of your disease	
2	Surviving your disease and living as long as possible	
3	Not feeling pain	
4	Keeping a normal amount of energy	
5	Ability to perform daily activities	
6	Ability to swallow	
7	Keeping a normal voice	
8	Keeping an unchanged appearance of your face	
9	Ability to chew normally	
10	Ability to taste normally	
11	Ability to smell normally	
12	Keeping a moist mouth	
13	Ability to breathe normally through mouth and nose	
14	Coping with anxiety	
15	Feeling not depressed	
16	Keeping trust in health care providers	
17	Not having financial worries with respect to your disease and its consequences	
18	Not being a burden to the others	
19	Not spending time in the hospital due to side effects	
20	Keeping a normal dental health	
21	Keeping a normal social life	
22	Having respect for your desires and dignity	
23	Keeping a normal sexuality	
24	Receiving information on the efficacy of your treatment and probability to survive in an open and sincere way	

#: number.

## Supplementary Materials

The following are available online at https://www.mdpi.com/2072-6694/12/12/3577/s1, Figure S1: flow chart of literature search; Table S1: Significant associations between selected issues and treating center (*n =* 109; *p*-value from chi-squared or Kendall Tau-b test); Table S2: Descriptive analysis of clustered issues and their distribution by treatment subgroups (*p*-value from Kruskal–Wallis test); Table S3: Distribution of issues analyzed separately as Likert-type data (frequencies, median, mode, range, IQR) and preferences (number, percentage) expressed by health care professionals; Table S4: Scoring criteria of issues according to the EORTC module development guidelines.
