# Supplementary material for: Assessing Preferences in Patients with Head and Neck Squamous Cell Carcinoma: Phase I and II of Questionnaire Development
Source: Cancers (Basel). 2020 Nov 30;12(12):3577. doi: 10.3390/cancers12123577 (PMC7760305; doi:10.3390/cancers12123577)
Supplement: Supplementary file 1 [file cancers-12-03577-s001.pdf]

Supplementary Materials: Assessing Preferences in Patients with Head and Neck Squamous Cell Carcinoma: Phase I and II of Questionnaire Development

Pierluigi Bonomo, Alice Maruelli, Calogero Saieva, Katherine Taylor, Susanne Singer, Zaira Patelli, Simon Rogers, Davide Mattavelli, Christian Simon, Florian Scotté, Thiago Bueno de Oliveira, Barbara Murphy, Bethany Andrews Rhoten, Umberto Tassini, Marie Fallon, Ourania Nicolatu Gatidis, Noam Yarom, Cristiana Bergamini and Paolo Bossi

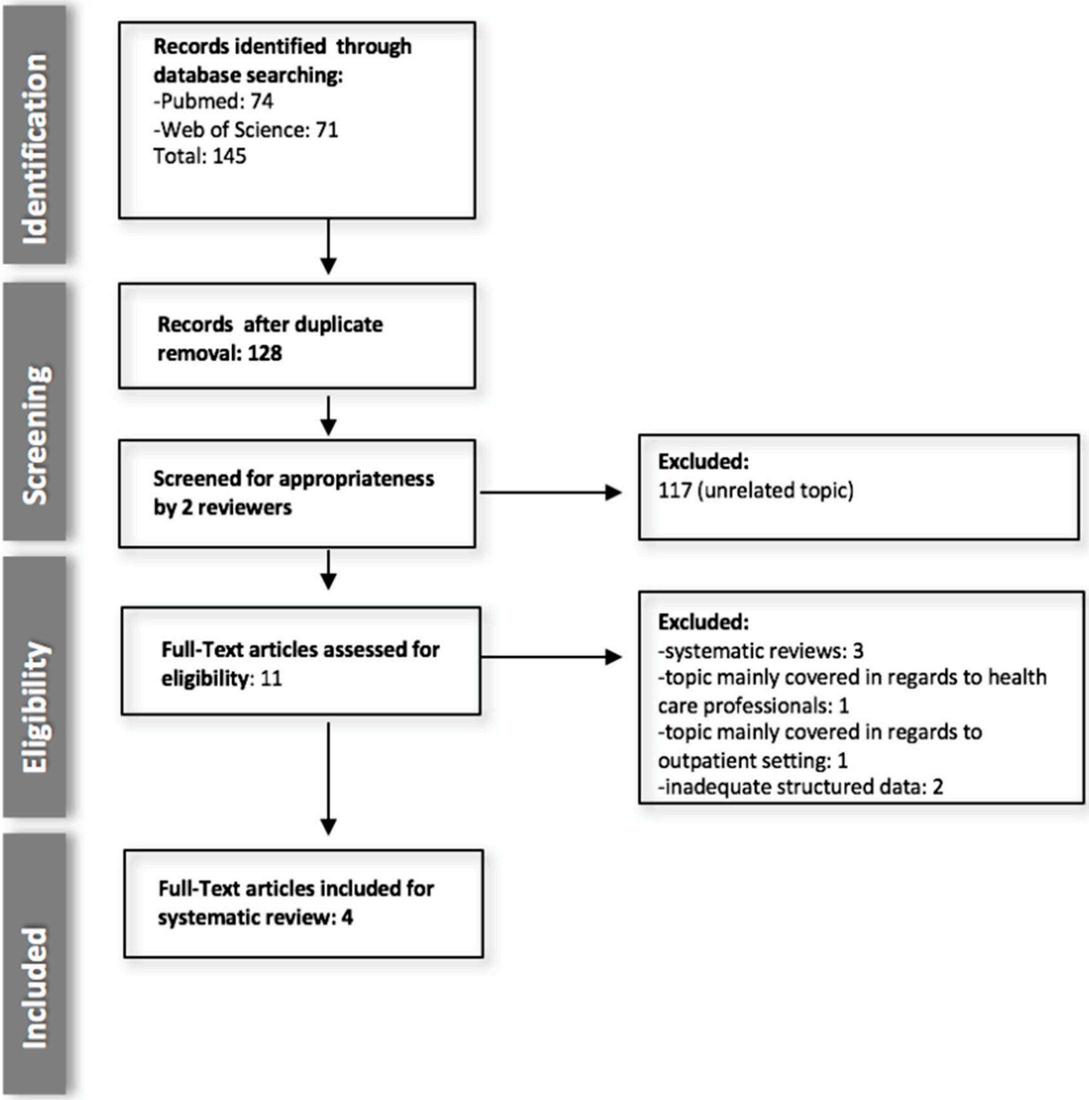

Figure S1. flow chart of literature search.

Table S1. significant associations between selected issues and treating center (*n* = 109; *p*-value from chi-square or Kendall tau B test).

Issue *n*	score	Brescia (*n* = 32) *n* (%)	Florence (*n* = 10) *n* (%)	Milan (*n* = 22) *n* (%)	Mainz (*n* = 10) *n* (%)	Sao Paulo (*n* = 15) *n* (%)	Nashville (*n* = 20) *n* (%)	*p*-value from Chi-square (Kendall tau B)
#1	2	0 (0)	2 (20)	0 (0)	0 (0)	0 (0)	0 (0)	0.008
	3	1 (3.1)	0 (0)	2 (9.5)	0 (0)	1 (6.7)	0 (0)	
	4	31 (96.9)	8 (80)	19 (90.5)	10 (100)	14 (93.3)	20 (100)	
#2	1	0 (0)	1 (10)	0 (0)	0 (0)	0 (0)	0 (0)	0.049
	2	0 (0)	0 (0)	0 (0)	0 (0)	1 (6.7)	0 (0)	
	3	2 (6.3)	0 (0)	5 (23.8)	1 (10)	2 (13.3)	0 (0)	
	4	30 (93.7)	9 (90)	16 (76.2)	9 (90)	12 (80)	20 (100)	
#3	1	1 (3.1)	1 (10)	1 (5.3)	1 (10)	0 (0)	1 (5)	0.018
	2	6 (18.8)	0 (0)	5 (26.3)	0 (0)	1 (6.7)	2 (10)	
	3	5 (15.6)	6 (60)	6 (31.6)	3 (30)	1 (6.7)	11 (55)	
	4	20 (62.5)	3 (30)	7 (36.8)	6 (60)	13 (86.6)	6 (30)	
#21	1	6 (18.8)	1 (10)	2 (10)	0 (0)	0 (0)	3 (15.8)	0.04
	2	6 (18.8)	2 (20)	8 (40)	0 (0)	1 (6.7)	8 (42.1)	
	3	7 (21.9)	3 (30)	5 (25)	4 (40)	9 (60)	6 (31.6)	
	4	13 (40.5)	4 (40)	5 (25)	6 (60)	5 (33.3)	2 (10.5)	
#22	1	7 (21.9)	0 (0)	2 (10.5)	1 (10)	1 (6.7)	1 (5.3)	0.043
	2	3 (9.4)	3 (30)	4 (21.1)	0 (0)	1 (6.7)	7 (36.8)	
	3	7 (21.9)	2 (20)	7 (36.8)	1 (10)	8 (53.3)	6 (31.6)	
	4	15 (46.8)	5 (50)	6 (31.6)	8 (80)	5 (33.3)	5 (26.3)	
#24	1	3 (9.4)	1 (10)	0 (0)	0 (0)	0 (0)	4 (21.1)	0.018 (0.017)
	2	3 (9.4)	2 (20)	5 (26.3)	0 (0)	3 (20)	6 (31.6)	
	3	7 (21.9)	2 (20)	9 (47.4)	2 (20)	4 (26.7)	7 (36.8)	
	4	19 (59.4)	5 (50)	5 (26.3)	8 (80)	8 (53.3)	2 (10.5)	
#25	1	2 (6.3)	0 (0)	0 (0)	1 (10)	0 (0)	0 (0)	0.063 (0.032)
	2	4 (12.5)	0 (0)	4 (20)	0 (0)	1 (6.7)	5 (26.3)	
	3	6 (18.8)	1 (10)	6 (30)	3 (30)	5 (33.3)	10 (52.6)	
	4	20 (62.4)	9 (90)	10 (50)	6 (60)	9 (60)	4 (21.1)	
#26	1	2 (6.5)	0 (0)	2 (10.5)	0 (0)	0 (0)	3 (15.8)	0.021
	2	5 (16.1)	0 (0)	5 (26.3)	0 (0)	1 (6.7)	3 (15.8)	
	3	10 (32.3)	3 (30)	5 (26.3)	0 (0)	9 (60)	8 (42.1)	
	4	14 (45.1)	7 (70)	7 (36.9)	10 (100)	5 (33.3)	5 (26.3)	
#28	1	4 (12.9)	0 (0)	0 (0)	0 (0)	0 (0)	0 (0)	0.025
	2	0 (0)	0 (0)	5 (25)	1 (10)	0 (0)	1 (5.6)	
	3	5 (16.1)	0 (0)	2 (10)	1 (10)	1 (6.7)	3 (16.7)	
	4	22 (71)	10 (100)	13 (65)	8 (80)	14 (93.3)	14 (77.7)	

#1: cure of disease; #2: survival (live as long as possible); #3: no pain; #21: social role; #22: normal dental health; #24: social life; #25: respect of desires and dignity; #26: intimacy; #28: being thoroughly and sincerely informed about treatments' efficacy and survival expectation.

Table S2. descriptive analysis of clustered issues and their distribution by treatment subgroups (*p*-value from Kruskal-Wallis test).

**Issues: #6 + #13 + #14**	**Curative (*n* = 30)**	**Follow-Up (*n* = 38)**	**Palliative (*n* = 20)**	**Cronbach's Alpha**
mean	10.7	9	10.3	0.68
SD	2.2	2.0	2.0	
min	3	4	6	
max	12	12	12	
p-value from Kruskal-Wallis test	all: *p* = 0.0001<br>follow-up vs palliative: *p* = 0.01<br>follow-up vs curative: *p* = 0.0001<br>palliative vs curative: *p* = 0.42			
**Issues: #16 + #24 + #26**	**Curative (*n* = 30)**	**Follow-Up (*n* = 36)**	**Palliative (*n* = 20)**	**Cronbach's Alpha**
mean	10.5	8.7	9.5	0.74
SD	1.9	2.6	2.5	
min	6	3	4	
max	12	12	12	
p-value from Kruskal-Wallis test	all: *p* = 0.009<br>follow-up vs palliative: *p* = 0.25<br>follow-up vs curative: *p* = 0.002<br>palliative vs curative: *p* = 0.13			

#6: ability to swallow; #13: normal breathing through mouth and nose; #14: burning mouth; #16: no depression; #24: social life; #26: intimacy.

Table S3. Distribution of issues analyzed separately as Likert-type data (frequencies, median, mode, range, IQR) and preferences (number, percentage) expressed by health care professionals.

**Score**	**1**		**2**		**3**		**4**		**5**		**6**		**7**		**8**		**9**		**10**	
	n	**%**	***n***	**%**	***n***	**%**	***n***	**%**	***n***	**%**	***n***	**%**	***n***	**%**	***n***	**%**	***n***	**%**	***n***	**%**
1	0	0	0	0	0	0	0	0	0	0	0	0	0	0	1	2.7	1	2.8	2	5.5
2	0	0	0	0	1	2.7	6	16.2	4	10.8	3	8.3	14	38.9	5	13.5	9	25	15	41.7
3	0	0	10	27	11	30.6	26	70.3	20	54	16	44.4	17	47.2	20	54	18	50	16	44.5
4	37	100	27	73	24	66.7	5	13.5	13	35.2	17	47.2	5	13.9	11	29.8	8	22.2	3	8.3
Total	37		37		36		37		37		36		36		37		36		36	
Median	4		4		4		3		3		3		3		3		3		2	
Mode	4		4		4		3		3		4		3		3		3		3	
Range	0		3–4		2–4		2–4		2–4		2–4		2–4		1–4		1–4		1–4	
IQR	0		1		1		0		1		1		1		1		1		1	
preference	33		26		28		9		26		29		10		19		10		3	
(%)	(89.2)		(70.3)		(77.8)		(24.3)		(70.3)		(80.6)		(27.8)		(51.4)		(27.8)		(8.3)	
Answer	**11**		**12**		**13**		**14**		**15**		**16**		**17**		**18**		**19**		**20**	
	n	**%**	***n***	**%**	***n***	**%**	***n***	**%**	***n***	**%**	***n***	**%**	***n***	**%**	***n***	**%**	***n***	**%**	***n***	**%**
1	5	13.9	2	5.5	0	0	1	2.8	1	2.8	1	2.8	2	5.5	2	5.5	1	2.8	0	0
2	21	58.3	10	27.8	9	25	16	44.4	15	41.7	13	36	4	11.2	6	16.7	11	30.5	13	36.1
3	9	25	19	52.8	17	47.2	14	38.9	14	38.8	18	50	14	38.9	15	41.7	15	41.7	18	50
4	1	2.8	5	13.9	10	27.8	5	13.9	6	16.7	4	11.2	16	44.4	13	36.1	9	25	5	13.9
Total	36		36		36		36		36		36		36		36		36		36	
Median	2		3		3		3		3		3		3		3		3		3	
Mode	2		3		3		2		2		3		4		3		3		3	
Range	1–4		1–4		2–4		1–4		1–4		1–4		1–4		1–4		1–4		2–4	
IQR	1		1		2		1		1		1		1		1		2		1	
preference	0		7		16		4		6		4		17		11		12		7	
(%)	(0)		(19.4)		(44.4)		(11.1)		(16.7)		(11.1)		(47.2)		(30.6)		(33.3)		(19.4)	
**Answer**	**21**		**22**		**23**		**24**		**25**		**26**		**27**				**28**			
	n	**%**	***n***	**%**	***n***	**%**	***n***	**%**	***n***	**%**	***n***	**%**	***n***		**%**		***n***		**%**	
**1**	1	2.8	3	8.3	5	13.9	1	2.8	1	2.7	3	8.1	3		8.1		2		5.4	

**2**	13	36.1	22	61.2	18	50	6	16.7	2	5.4	13	35.2	13	35.2	3	8.1
**3**	14	38.9	8	22.2	13	36.1	21	58.3	15	40.5	15	40.5	13	35.2	10	27.1
**4**	8	22.2	3	8.3	0	0	8	22.2	19	51.4	6	16.2	8	21.5	22	59.4
**Total**	36		36		36		36		37		37		37		37	
**Median**	3		2		2		3		4		3		3		4	
**Mode**	3		2		2		3		4		3		2		4	
**Range**	1–4		1–4		1–3		1–4		1–4		1–4		1–4		1–4	
**IQR**	1		1		1		0		1		1		1		1	
**preference**	5		1		2		8		21		4		4		17	
**(%)**	(13.9)		(2.8)		(5.6)		(22.2)		(56.8)		(10.8)		(10.8)		(45.9)	

IQR: interquartile range.

Table S4. scoring criteria of issues according to the EORTC module development guidelines.

Criteria	Scoring Criteria	Threshold	Score for Inclusion
1	Mean of patients' Likert score	> 2.0	1
2	Mean of relevance to a specific patients' subgroup (curative intent subgroup; palliative intent subgroup; follow-up subgroup)	> 2.5	1
3	>20% of the patients said yes to inclusion	> 20%	1
4	>30% of HCP said yes to inclusion	> 30%	1

HCP: health care professionals.

Publisher's Note: MDPI stays neutral with regard to jurisdictional claims in published maps and institutional affiliations.

© 2020 by the authors. Licensee MDPI, Basel, Switzerland. This article is an open access article distributed under the terms and conditions of the Creative Commons Attribution (CC BY) license (http://creativecommons.org/licenses/by/4.0/).
